# Recognising the role of ophthalmic nurses and allied ophthalmic personnel in eye care

**Published:** 2020-12-31

**Authors:** Elmien Wolvaardt, Michelle Hennelly

**Affiliations:** 1 Editor: *Community Eye Health Journal*, International Centre for Eye Health, London School of Hygiene & Tropical Medicine, London, UK.; 2 MSc Programme Director in Clinical Optometry: Division of Optometry and Visual Science, City, University of London, UK.

**Ophthalmic nurses, ophthalmic clinical officers and other allied ophthalmic personnel are at the forefront of the eye health workforce, particularly in low-resource settings where there is a shortage of ophthalmologists.**

**Figure:**
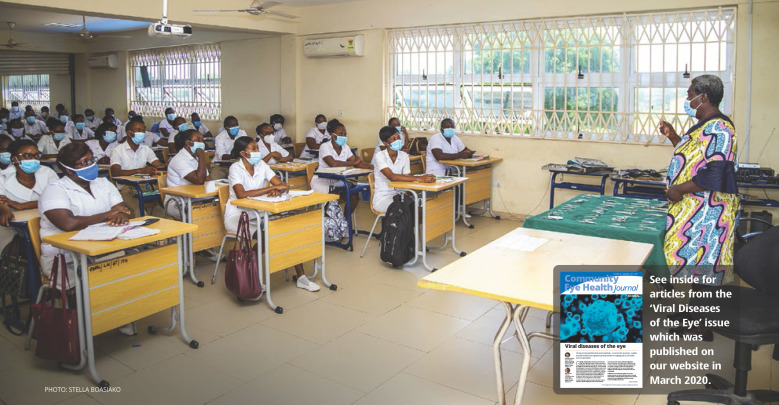
Nurses undergoing training at the Ophthalmic Nursing School in Kurle-Bu, Ghana. Windows are open to improve air circulation and both lecturer and students are wearing face masks to reduce droplet transmission. **GHANA**

COVID-19 has overtaken our lives in so many ways over the last year. Eye care providers worldwide are having to find new ways to deliver eye care in the midst of the global COVID-19 pandemic. While news of vaccines bring hope for the future, the pandemic is far from over, with many services still closed or struggling.

In such uncertain times, it is more important than ever that eye teams work together. Regular meetings and agreed protocols and processes are important components of teamwork, but just as vital is that we understand who our team members are, know what they do, and take time to listen to them and value their contributions to the smooth functioning of eye services.

In recognition of the World Health Organization's Year of the Nurse and Midwife in 2020, we have dedicated the first section of this issue to reflecting on the vital contributions of ophthalmic nurses and allied health personnel in delivering integrated, people-centred eye care. In the second part of the issue, we also take a detailed look at different viral infections of the eye and how to detect, diagnose and manage them, with a detailed review of antiviral treatment for common eye conditions.

We hope you enjoy the issue. Remember that you can now have *Community Eye Health Journal* articles delivered directly to your phone as soon as they become available - download our app from Google Play (**bit.ly/CEHJ-Android**) or the App Store (**bit.ly/CEHJ-ios**).

